# Matrix metalloproteinase-2 is a consistent prognostic factor in gastric cancer

**DOI:** 10.1038/sj.bjc.6603041

**Published:** 2006-03-14

**Authors:** F J G M Kubben, C F M Sier, W van Duijn, G Griffioen, R Hanemaaijer, C J H van de Velde, J H J M van Krieken, C B H W Lamers, H W Verspaget

**Affiliations:** 1 Department of Gastroenterology and Hepatology, Leiden University Medical Centre, Building 1, C4-P012, PO Box 9600, 2300 RC Leiden, The Netherlands; 2 TNO Quality of Life, Biomedical Research, Leiden, The Netherlands; 3 Department of Oncologic Surgery, University Medical Centre Leiden, Leiden, The Netherlands; 4 Department of Pathology, University Medical Centre Nijmegen, Nijmegen, The Netherlands

**Keywords:** gelatinases, MMP-7, MMP-8, MMP-9, TIMP, tissue inhibitor of metalloproteinases

## Abstract

In a pioneer study, we showed 10 years ago that enhanced tissue levels of the matrix metalloproteinases (MMPs) MMP-2 and MMP-9 in gastric cancers, as determined by zymography, were related with worse overall survival of the patients. To corroborate these observations, we now assessed MMP-2 and MMP-9 with new techniques in an expanded group of gastric cancer patients (*n*=81) and included for comparison MMP-7, MMP-8 and the tissue inhibitors of MMPs, TIMP-1 and -2. All MMPs and TIMP-1 were significantly increased in tumour tissue compared to normal gastric mucosa. Matrix metalloproteinase-7, -8 and -9, and the TIMPs showed some correlations with the clinicopathologic parameters TNM, WHO and Laurén classification, but their levels were not related with survival. Regardless of the determination method used, that is, enzyme-linked immunosorbent assay or bioactivity assay, an enhanced tumour MMP-2 level did not show a significant correlation with any of the clinicopathological parameters, but was confirmed to be an independent prognostic factor in gastric cancer.

A decade ago, we were the first to report that the levels of matrix metalloproteinase (MMP)-2 and MMP-9 in human gastric carcinoma tissues were enhanced and related to the survival of the patients, using a simple but laborious zymography technique in a relatively small group of patients (Sier *et al*, 1996). Matrix metalloproteinases are believed to play an important role in carcinogenesis via the degradation and remodelling of tumour surrounding extracellular matrix, which could explain the association with survival (Zucker *et al*, 2000; McCawley and Matrisian, 2001; Polette *et al*, 2004). We concluded that measuring MMPs could have clinical value as indicators for gastric carcinoma patients who needed adjuvant therapy and that inhibitors of MMPs might be useful for therapeutic intervention. Several accomplishments have been made since. The prognostic value of MMPs for gastric carcinoma patients has been confirmed in several other studies (Allgayer *et al*, 1998; Zhang *et al*, 2003), and clinical trials testing the effect of MMP inhibitors for patients with various types of cancer were performed, with variable success (Zucker *et al*, 2000; Bramhall *et al*, 2002).

In general, MMPs are secreted as inactive pro-enzymes, activated by proteolytic cleavage, and controlled in their activity by interaction with inhibitors. Disturbances in these processes are of eminent importance in tumour invasion and metastasis (McCawley and Matrisian, 2001; Polette *et al*, 2004). In the present more comprehensive study, we extended our MMP analyses in the same group of patients and compared the results with those obtained with a new and more recent group of patients. Furthermore, instead of zymography, which identifies isoforms, we now used recently established quantitative bioactivity assays (BIAs) and specific antigen enzyme-linked immunosorbent assays (ELISAs) for MMP-2 and MMP-9. Moreover, we compared the prognostic value of MMP-2 and MMP-9 with those of MMP-7 and MMP-8 and expanded the study by determination of the inhibitors TIMP-1 and TIMP-2. In addition, because of the increasing age of the patients and the length of the follow-up, we now used tumour-associated survival.

## PATIENTS, MATERIALS AND METHODS

### Patients and study design

Fresh tissue specimens of 81 patients (21 female and 60 male subjects, mean age 65.9 years, range 35.10–91.33), who underwent resection for primary gastric adenocarcinoma at the Department of Oncologic Surgery of the Leiden University Medical Centre, were collected prospectively. Immediately after resection, fresh samples from the mid-central, non-necrotic part of the carcinoma and/or from distant normal mucosa, taken approximately 10 cm from the tumour, were snap frozen and stored at −70°C until extraction, to be used for research purposes. Various clinicopathological data were evaluated or collected from patient files. All carcinomas were classified according to the TNM classification (Hermanek and Sobin, 1992) and localisation and also diameter of the tumour, differentiation grade, WHO, Borrman, and Laurén classification, as well as the presence of intestinal metaplasia in the normal gastric mucosa, as revised by a gastroenterologist (FK) and a pathologist (JvK). All patients entered the study at operation date, and the patient's time experience ended in the event of death or, when still alive, at the common closing date. The minimal follow-up was 33 months with a decreasing overall survival according to TNM stage, that is, from TNM I (52.2%, *n*=23), to TNM II (26.9%, *n*=26), to TNM III (28%, *n*=25), and to TNM IV (0%, *n*=7).

### Tissue preparation and protein concentration

Homogenisation of tissue specimens and determination of protein concentrations were performed as described previously (Sier *et al*, 1996).

### Metalloproteinase-2 and -9 activity assays

Quantitative gelatin zymography and BIAs for MMP-2 and MMP-9 were carried out as described before (Sier *et al*, 1996; Hanemaaijer *et al*, 2000). Active and activatable (pro) MMP-2 and MMP-9 were determined with the BIA in 96-well plates, coated with monospecific antibodies to the MMPs, sample/standard incubation overnight and detection by modified MMP-sensitive pro-urokinase in combination with peptide substrate S-2444 and measurement of absorbance change at 405 nm over time. Activation of pro-MMPs was achieved by incubation with *p*-aminophenyl-mercuric acetate.

### Enzyme-linked immunosorbent assay for MMP-2, MMP-7, MMP-8, MMP-9, TIMP-1 and TIMP-2

Antigen levels of MMP-2 and MMP-9 were determined using previously described ELISAs (Hanemaaijer *et al*, 1998). In brief, the same catching antibodies were used as for the BIAs. Next, appropriate dilutions of tissue homogenates were incubated overnight at 4°C. Immunodetection of MMP-2 and MMP-9 was performed directly or indirectly with in-house anti-MMP-2 and -MMP-9 biotinylated-polyclonal antibodies. Avidin–horseradish peroxidase and 3,3′,5,5′ tetramethyl benzidine were used for the colouration reaction. The respective amounts of MMP-2 and MMP-9 were calculated from standard curves. The concentrations of MMP-7, TIMP1 and TIMP-2 antigens were determined using commercial ELISAs according to the manufacturer's instructions (R&D Systems Europe, Abingdon, UK). The amount of MMP-8 was measured using a previously described ELISA (Bergmann *et al*, 1989).

### Statistical analysis

Differences between normal and tumour values for all parameters were calculated using the Wilcoxon signed rank test. For the survival analyses, the clinicopathological parameters were dichotomised as described previously unless indicated elsewhere. Cutoff points for MMP data were optimised or medians were used. Univariate and multivariate survival analyses were performed with the Cox proportional hazards model, using the SPSS Windows Release 12.0.1. statistical package (2004, SPSS Inc., Chicago, IL, USA). Multivariate survival analyses were performed using the Cox proportional hazards method by separately adding the significant MMP variables to the dichotomised clinicopathological parameters. Overall and tumour-related survival curves were constructed using the method of Kaplan and Meier including the Log-rank test. Differences were considered significant when *P*⩽0.05.

## RESULTS

Although quantitative zymography is a reliable and sensitive technique to identify active and latent isoforms of MMP-2 and MMP-9, it is a laborious assay to perform. Therefore, we compared the previously obtained zymography data for MMP-2 and MMP-9 with the results from more practicable and sophisticated immunoassays, that is, BIAs and ELISAs. Table 1 shows an overview of the correlation coefficients and *P*-values for the different assays (samples *n*=100). The total zymography data, which consist of the sum of active and pro-form bands, correlated significantly with the total BIA and ELISA levels for both MMP-2 (0.312<*ρ*<0.533, *P*⩽0.003) and even better for MMP-9 (0.558<*ρ*<0.817, *P*<0.001). The latent pro-forms of MMP-2 and MMP-9, separately detected by zymography and BIA, also correlated significantly. No correlation between both assays was found, however, for active MMP-2 or MMP-9, indicating that the active isoform as identified by the very sensitive zymography is not necessarily functionally active in the less-sensitive BIA, probably through interaction with inhibitors.

The levels of MMP-2 and MMP-9 as detected with the BIAs and ELISAs in normal mucosa and tumour tissue in the expanded group of 81 gastric carcinoma patients are shown in Table 2. Carcinomas contained significantly higher MMP-2 and MMP-9 levels in antigen as well as activity than adjacent normal tissue. Particularly remarkable is the presence of more active MMP-2, but not of active MMP-9, in the tumour tissue homogenates. The most impressive enhancement (>20-fold) in carcinomas compared to normal tissue, however, was noted for MMP-7 (Table 2). Matrix metalloproteinase-8 and TIMP-1 were also significantly increased, whereas tumour TIMP-2 levels were found not to be enhanced. Interestingly, a striking difference was observed in the correlation between the primary MMP–TIMP interactor antigen levels, that is, MMP-9 with TIMP-1 (*ρ*=0.358, *P*<0.0005) and MMP-2 with TIMP-2 (*ρ*=0.085, NS).

**Figure 1:**
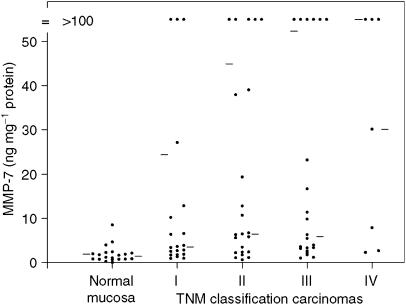
Relation between MMP-7 antigen levels and TNM classification in gastric carcinomas. The mean and median for the subgroups are indicated by bars on, respectively, the left- and right-hand side of each column.

The levels of MMPs and TIMPs were also evaluated for correlation with all the clinicopathological parameters. Tumour levels of MMP-2, TIMP-1 and TIMP-2 did not show significant correlations with any of these parameters. The mean MMP-7 levels increased stepwise with TNM classification (Figure 1) and were significantly enhanced in Laurén's intestinal-type carcinomas compared to diffuse or mixed types (56±16 *vs* 34±22, *P*<0.02). Matrix metalloproteinase-8 levels were enhanced in Laurén's intestinal-type tumours (402±72 *vs* 178±29 ng mg^−1^ protein, *P*<0.006) and differentiated tumours (393±67 *vs* 163±29 ng mg^−1^ protein, *P*<0.002) according to the WHO classification. Matrix metalloproteinase-9 levels showed a similar enhancement for Laurén's intestinal-type carcinomas (BIA total activity 140 *vs* 99 U mg^−1^ protein, *P*<0.02; ELISA 29 *vs* 17 ng mg^−1^ protein, *P*<0.01) and differentiated tumours (BIA total activity 133±13 *vs* 104±16 U mg^−1^ protein, NS; ELISA 28±3 *vs* 17±3 ng mg^−1^ protein, *P*<0.02). Matrix metalloproteinase-9 total activity showed a stepwise decrease with TNM classification, which did not reach significance (I, 145±27; II 127±18; III, 120±20; IV, 114±28 U mg^−1^ protein).

**Figure 2:**
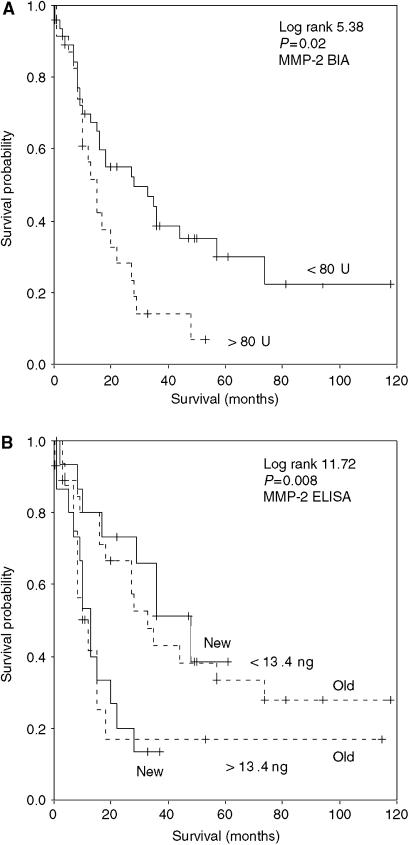
Kaplan–Meier tumour-related overall survival curves for (**A**) MMP-2 BIA total activity, (**B**) MMP-2 ELISA old *vs* new gastric cancer patient groups, with the cutoff levels from the Cox analyses.

For tumour-associated survival analyses, all MMP and TIMP parameters in tumour homogenates were evaluated for optimal cutoff points using the log rank test. Significant cutoffs were only found for the MMP-2 levels by BIA and ELISA (Figures 2A and B). No significant association for MMP-7, MMP-8, MMP-9, TIMP-1 and TIMP-2 with tumour-associated survival was found according to stepwise univariate Cox analyses and thus the medians were used (hazard ratio and 95% confidence interval ranges of the median levels varied from 0.801 to 1.257 and from 0.445 to 2.307, respectively). High MMP-2 levels determined by BIA as well as ELISA were significantly associated with worse survival, but in multivariate analyses with the clinicopathological parameters, only the MMP-2 ELISA kept its independent prognostic value (Table 3). The consistent prognostic relevance of MMP-2 is underlined by Figure 2B, in which the old group of patients (*n*=50) and the more recent patients group (*n*=31) are independently subdivided based on a low or high MMP-2 antigen content of the carcinoma, using the same cutoff value. Similar results were obtained with the BIA data (not shown).

## DISCUSSION

The present study corroborates our previous finding of increased MMP-2 in gastric cancer. The high MMP-2 antigen and activity levels were significantly associated with worse survival according to univariate Cox proportional hazards analysis. In the multivariate analysis, including a broad selection of clinical parameters, the MMP-2 antigen level kept its independent prognostic value, but the significance for the MMP-2 BIA activity level of the carcinomas was lost. The optimal cutoff point for MMP-2 antigen calculated for survival prognosis in the old group of patients was similarly predictive in the new group of patients, indicating the strength of MMP-2 as a prognostic indicator for gastric carcinoma patients. The notion that MMP-2 is a valuable indicator of gastric cancer progression and prognosis is supported by immunohistochemical, zymography and mRNA studies showing that MMP-2 is associated with tumour invasion, lymph node metastasis and survival (Allgayer *et al*, 1998; Mönig *et al*, 2001; Chuanzhong *et al*, 2002; Kabashima *et al*, 2002; Liu *et al*, 2002a; Elnemr *et al*, 2003; Yokoyama *et al*, 2004; Ji *et al*, 2005). The value of MMP-2 as an independent prognostic marker for gastric carcinomas is underscored by our observation that MMP-9, MMP-7, MMP-8, TIMP-1 and TIMP-2 have no prognostic relevance.

Matrix metalloproteinase-9 levels were enhanced in some clinicopathological subgroups of gastric cancer, that is, according to the Laurén classification and for WHO differentiation grade. The association between MMP-9 and early stages of gastric carcinoma, as shown before (Torii *et al*, 1998; Kabashima *et al*, 2000), was also present in our study. In contrast to our previous findings, high MMP-9 levels did not show a significant correlation with survival and also not for the ratio MMP-9/TIMP-1 (data not shown) as recently suggested (Zhang *et al*, 2003). One obvious explanation for the discrepancy with our previous data is the small number of patients in the study. However, the relatively high MMP-9 levels in early gastric carcinomas also might affect the relation between MMP-9 and prognosis, especially in our extended follow-up study using tumour-related survival.

Matrix metalloproteinase-7, MMP-8, TIMP-1 and TIMP-2 were included in the present study as comparisons to evaluate the prognostic strength of MMP-2 and MMP-9. Matrix metalloproteinase-7 was selected because MMP-7 production in various types of carcinomas has predominantly been found in tumour cells and because MMP-7 was recently suggested as potential marker for gastric carcinoma (Liu *et al*, 2002b). Although enhanced levels were found in the different carcinoma subgroups, for example, TNM stage and Laurén's intestinal type, there was no correlation between high MMP-7 levels and patients survival. This contrasts in part with several other studies reporting not only a clear association between MMP-7 expression and gastric cancer progression but also with survival (Liu *et al*, 2002b; Ajisaka *et al*, 2004). Essential differences with our study are, however, that the latter studies were carried out using immunohistochemistry, focusing on MMP-7-expressing carcinoma cells at the invasive front, whereas our ELISA antigen values were derived from representative overall parts of the tumours. Matrix metalloproteinase-8, like MMP-9, is mainly present in neutrophils in carcinomas. Therefore, the expected correlation in presence of MMP-8 and MMP-9 was confirmed by the high correlation between both antigen levels (*ρ* 0.810, *P*⩽0.001, *n*=158), and the similar distribution according to the different cancer subgroups. The lack of correlation with survival was, therefore, not surprising in this study, as described by others before (Yokoyama *et al*, 2004).

The levels of TIMP-1 were significantly enhanced in cancer tissue, but the previously found association between TIMP-1 levels in sections or homogenates from gastric cancer tissue with survival (Joo *et al*, 2000; Yoshikawa *et al*, 2001) was not observed in our group of patients. However, our group contained relatively less patients with advanced TNM stages, which could account for the different results compared with these former studies. In contrast to what was expected from *in vitro* studies (Koyama, 2004), we did not find differences between TIMP-2 levels in normal and cancerous tissue. Also, the levels between different tumour subgroups did not vary, indicating a rather constitutive expression of this inhibitor. As TIMP-2 immunohistochemical staining combined with *in situ* hybridisation experiments detected the expression of TIMP-2 in gastric cancer tissue, primarily in peritumoral stromal cells rather than in malignant cells (Joo *et al*, 2000), we conclude that the localisation of TIMP-2 within the cancerous tissue might be of crucial importance but apparently not the total amount of the inhibitor. The recently suggested role for TIMP-2 in the activation of pro-MMP-2 (Itoh *et al*, 2001), combined with the different cell types involved in the expression of MMP-2 and its main inhibitor TIMP-2 in gastric carcinoma, indicate the importance of local cell–cell and molecule–molecule interactions in the activation process. This is particularly noticeable from our finding that there is no correlation between MMP-2 and TIMP-2 levels in the tissue homogenates, where the increase in MMP-2 outbalances that of TIMP-2, resulting in an increased net MMP-2 activity in the tumours, an observation which can only be made by using the BIA. This process was not observed with MMP-9 and TIMP-1, where a more balanced increase was found in the tumours.

Although many *in vitro* studies, animal models and clinical studies clearly showed that MMPs are indeed involved in a number of critical steps during tumour growth and invasion, most synthetic MMP inhibitors, designed as anticancer agents, failed to improve patients outcome in clinical trials (Zucker *et al*, 2000), showing that our understanding of the working mechanisms of MMPs in tumour biology is still poor. Coincidentally, gastric cancer appeared to be one of the few cancers for which a significant survival benefit from therapy with a matrix metalloproteinase inhibitor has been described (Bramhall *et al*, 2002). Recent studies indicate that proteolytic MMP activity is involved in the uncovering or release of specific sites from macromolecules in the extracellular matrix (McCawley and Matrisian, 2001; Polette *et al*, 2004), which at least *in vitro* leads to various biological activities. Our study shows that an enhanced MMP-2 level is consistently and more strongly associated with prognosis of gastric cancer patients than other MMPs or TIMPs. This association might be caused by the noninvasion-related activities of MMPs, like cytokine release/activation, which makes MMP-2 in our opinion an important player in gastric cancer, deserving further investigation. Finally, differences in the association of the other MMPs and TIMPs with gastric cancer survival between our study and other reports, as mentioned above, might be related to differences in genetic background, that is, Caucasian *vs* Asian, which is currently under study.

## Figures and Tables

**Table 1:**
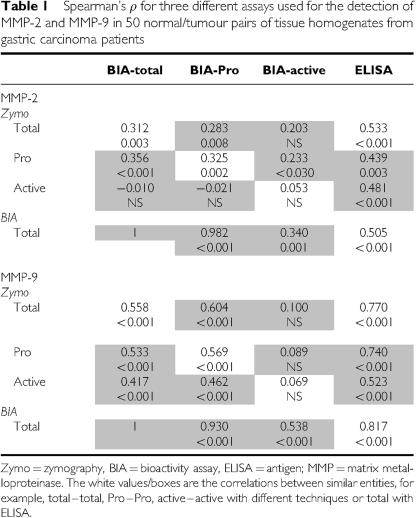
Spearman's *ρ* for three different assays used for the detection of MMP-2 and MMP-9 in 50 normal/tumour pairs of tissue homogenates from gastric carcinoma patients

**Table 2:** Antigen levels (ng mg^−1^ protein) of MMP-2, MMP-7, MMP-8 and MMP-9 and of inhibitors TIMP-1 and TIMP-2 in normal mucosa and carcinoma of 81 patients with gastric cancer

	**Mucosa**	**Carcinoma**	***P*-value**
*MMP-2*			
Antigen	4.7±0.4	17.0±2.0	⩽0.001
Total activity^a^	81.1±23.6	185.7±45.5	⩽0.001
Pro-form^a^	78.9±23.6	181.1±45.3	0.001
Active^a^	2.3±0.5	4.7±1.1	0.02
*MMP-9*			
Antigen	9.0±0.9	24.7±2.3	⩽0.001
Total activity^a^	67.5±6.0	128.8±11.3	⩽0.001
Pro-form^a^	59.9±5.6	117.1±10.1	⩽0.001
Active^a^	7.6±1.5	9.5±2.1	NS
MMP-7	2.0±0.5	47.1±12.4	0.002
MMP-8	95±12	319±47	⩽0.001
TIMP-1	8.0±0.8	16.9±1.3	⩽0.001
TIMP-2	5.9±0.2	6.3±0.4	NS

Mean±s.e.m.

Bioactivity assay levels of MMP-2 and MMP-9 are expressed as units per mg protein.

a As determined by BIA.

MMP=matrix metalloproteinase; BIA=bioactivity assay; TIMP=tissue inhibitors of MMP.

**Table 3:** Uni- and multivariate Cox's proportional hazards analyses of MMP-2, determined by ELISA and BIA, and clinicopathological parameters in relation to the overall tumour-related survival of 81 patients with gastric cancer

		**Univariate**			**Multivariate**		
**Parameter**	***n***	**HR**	**CI 95%**	***P***	**HR**	**CI 95%**	***P***
*Gender*							
Male *vs* female	60/21	1.384	0.768–2.494	NS	1.767	0.935–3.342	NS
*Age*							
Median (66 years)	40/41	1.313	0.764–2.255	NS	1.467	0.775–2.774	NS
*TNM*							
I	23/81	1	—		1	—	
II	26/81	3.133	1.360–7.222	0.007	4.001	1.510–10.60	0.005
III	25/81	3.021	1.305–6.991	0.010	3.557	1.290–9.813	0.014
IV	7/81	7.387	2.495–21.86	0.000	20.416	4.992–83.49	0.000
*Laurén*							
Diffuse/mixed *vs* intestinal	30/50	0.889	0.516–1.531	NS	1.152	0.353–3.756	NS
*WHO differentiation*							
Well *vs* poor	54/26	1.133	0.650–1.975	NS	1.270	0.370–4.363	NS
*Borrmann*							
I+II *vs* III+IV	55/24	1.118	0.609–2.053	NS	0.761	0.386–1.502	NS
*Localisation*							
Cardia *vs* rest	36/45	0.573	0.330–0.993	0.034	0.330	0.159–0.682	0.003
*Diameter tumour*							
<5 *vs* >5 cm	47/34	1.048	0.608–1.808	NS	0.622	0.337–1.149	NS
*Eosinophils*							
Few *vs* many	56/24	1.035	0.568–1.886	NS	1.743	0.806–3.766	NS
*Intestinal metaplasia*							
Absent *vs* present	39/42	0.490	0.280–0.858	0.013	0.706	0.379–1.315	NS
*Carcinoma*							
MMP-2 ELISA							
<13.4 *vs* >13.4 ng mg^−1^ protein	45/31	2.611	1.455–4.686	0.001	2.620	1.249–5.494	0.011
MMP-2 BIA							
<80 *vs* >80 U mg^−1^ protein	49/23	1.974	1.089–3.577	0.025	1.493	0.655–3.404	NS

HR=hazard ratio; CI=confidence interval.
